# BMP-2 in Orthopedic Trauma and Limb Reconstruction: Current Evidence, Limitations, and Future Directions

**DOI:** 10.3390/biomedicines14102267

**Published:** 2026-10-07

**Authors:** Muhammad Ade Junaidi, Filberto Budhy, Mulki Hakam, Bany Faris Amin, Muhammad Orri Baskoro

**Affiliations:** 1 Trauma Division, Department of Orthopedics and Traumatology, Dr. Cipto Mangunkusumo Hospital, Faculty of Medicine Universitas Indonesia, Jakarta Pusat 10430, Indonesia; 2 Department of Orthopedics and Traumatology, Dr. Cipto Mangunkusumo Hospital, Faculty of Medicine Universitas Indonesia, Jakarta Pusat 10430, Indonesia

**Keywords:** bone morphogenetic protein-2, BMP-2, orthopedic trauma, fracture healing, bone defect, limb reconstruction, bone regeneration

## Abstract

**Background/Objectives**: Recombinant human bone morphogenetic protein-2 (rhBMP-2) is an osteoinductive agent increasingly used in many orthopedic cases such as fracture healing, nonunion, critical-sized bone defects, and limb reconstruction cases. However, its efficacy and safety remain controversial especially regarding its optimal dosing, delivery, and adverse effect concerns. This narrative review aims to summarize the rhBMP-2 evidence in orthopedic trauma and limb reconstruction. **Methods**: A literature search was conducted on PubMed/MEDLINE, Scopus, and Web of Science up to August 2026. We prioritized meta-analyses, systematic reviews, randomized controlled trials, and clinical studies. **Results**: rhBMP-2 induces osteogenic differentiation, endochondral ossification, and bone regeneration through canonical Smad-dependent and non-canonical pathways. Current evidence suggests a potential use of rhBMP-2 as an adjunct in selected open fractures, delayed union and nonunion, and complex reconstruction patients, although the results are still heterogeneous. Some studies reported fewer secondary interventions in open tibial fractures, while others failed to reproduce a consistent advantage over standard care or autograft. Hydroxyapatite-based delivery systems have recently demonstrated promising union rates at lower total rhBMP-2 doses, suggesting that the carrier and delivery method may determine the effect. Currently available studies carry clear weaknesses: small samples, few adequately powered trials, heterogeneous indications, no established optimal dose, high cost, and complications that include inflammation and ectopic bone formation. Controlled-release scaffolds, heparin-based microparticles, biomimetic carriers, and gene- or mRNA-based delivery are future research directions currently under investigation. **Conclusions**: rhBMP-2 is a promising adjunct that may benefit selected high-risk patients, but it does not replace stable fixation, infection control, soft-tissue management, or autologous grafting. Further trials are needed to define appropriate patient selection, the minimum effective dose, and optimal delivery systems.

## 1. Introduction

Fractures and their disability account for a big part of the global injury burden, with an estimated 178 million new fractures worldwide every year and 25.8 million years lived with disability [1]. This disability is caused by delayed union, nonunion, and critical-sized defects, which often cluster in patients who sustain high-energy trauma, extensive soft-tissue injury, infection, poor vascularity, or segmental bone loss. Treating these cases often needs repeated surgery, months of rehabilitation, and complex limb reconstruction, at considerable functional and socioeconomic cost [2].

Fracture management depends on stable fixation, infection control, vascularized soft-tissue coverage, and restored alignment; however, the bone defect often determines the need for reconstruction, whether that means autologous bone graft, distraction osteogenesis, vascularized bone transfer, or a structural allograft [3]. Donor-site morbidity, treatment duration, and results also vary from patient to patient. Those limits explain the interest in biological augmentation, which combines osteogenic cells, osteoconductive scaffolds, and osteoinductive growth factors.

Bone morphogenetic protein-2 (BMP-2) has drawn interest as an osteoinductive agent in orthopedic cases. This protein drives osteogenic differentiation and de novo bone formation and has been investigated as an adjunct in fracture healing and bone defect reconstruction, with the BESTT trial demonstrating that rhBMP-2 added to standard treatment of open fractures has shown reduced secondary interventions and improved fracture healing [4]. However, many studies have shown variable efficacy, and concerns regarding its optimal dose, local inflammation, ectopic bone formation, wound complications, delivery methods, and cost have limited the broader application of rhBMP-2 [5,6]. Patient conditions such as diabetes and osteoporosis, which impair bone regeneration, may also influence the effectiveness of rhBMP-2 and, consequently, the delivery systems required [7].

Accordingly, current research has shifted toward optimizing patient selection, dose, and delivery systems to maximize the regenerative potential of BMP-2 while minimizing its adverse effects. Novel biomaterial carriers, controlled-release platforms, and gene- or RNA-based delivery strategies are being investigated to provide more localized and sustained BMP-2 activity. This narrative review gathers current evidence on BMP-2 in orthopedic trauma and limb reconstruction, especially relating to its clinical efficacy, dose/carrier considerations, safety, limitations, and future directions for safer and more effective use.

## 2. Methods

A literature search was conducted using PubMed/MEDLINE, Scopus, and Web of Science covering research published through August 10, 2026. Search strings combined “bone morphogenetic protein-2,” “BMP-2,” and “rhBMP-2” with “orthopedic trauma,” “fracture,” “bone defect,” “nonunion,” “delayed union,” “osteomyelitis,” “limb reconstruction,” and “bone regeneration.” Titles and abstracts were screened independently by two authors (M.A.J. and B.F.A.) who then assessed the full texts of potentially relevant articles. We also screened the reference lists of retrieved articles for eligible studies that the database search had missed. 

The authors performed study selection related to the biological mechanism of BMP-2, its clinical applications, safety profile, dosing, and delivery in fracture healing and post-traumatic bone reconstruction. The following studies were prioritized: randomized controlled trials, prospective and retrospective clinical studies, systematic reviews and meta-analyses, and translational work with direct clinical bearing. We included publications primarily from the last 5 to 10 years, while older sources were included if they documented the development of BMP-2 or supply pivotal clinical evidence. When the same publication was retrieved from more than one database, it was considered only once. Language restriction was not applied during the database search. Publications were excluded from the clinical discussion when they did not evaluate BMP-2, were unrelated to fracture healing or post-traumatic limb reconstruction, or did not provide information relevant to the predefined scope of the review. Given the narrative nature of this review, the authors performed no formal meta-analysis or quantitative evidence synthesis.

## 3. Mechanisms of BMP-2 

### 3.1. Structure

Bone morphogenetic proteins are members of the transforming growth factor-beta (TGF-β) superfamily, with approximately 20 BMPs identified. BMP-2 is initially synthesized as an inactive preprotein and becomes biologically active after proteolytic cleavage by proprotein convertases, producing a mature 115-amino-acid protein [8,9]. This cleaved BMP-2 protein is then secreted in an autocrine or paracrine manner from cells and can bind to osteoblast and osteoclast receptors [10]. After its release, this protein is further processed by proteases and signals through heteromeric receptor complexes made of type II and type I serine-threonine kinase receptors, and BMP-2 promotes receptor dimerization or recruitment at the cell surface [8,11]. The BMP monomer (Figure 1) consists of seven conserved cysteine residues within its core with six of them positioned in the C-terminal region. Two pairs of antiparallel β-strands form the characteristic “fingers,” which extend from a central α-helix or “wrist” region. These structural monomers then form concave and convex surfaces that introduce specific interactions with BMP receptors [12].

### 3.2. Signaling Pathway

BMP-2 regulates osteogenesis, cell differentiation, and tissue development through two intracellular cascades initiated by membrane receptor complexes, known as the canonical (Smad-dependent) and non-canonical (Smad-independent) pathways (Figure 2) [13]. Upon binding to the type II receptor, BMP-2 will activate BMPRII phosphorylation of type I BMP receptors (BMPRIa/BMPRIb), which will activate two parallel downstream cascades. The canonical Smad1/5/8 pathway involves receptor-mediated phosphorylation of Smad1, Smad5, and Smad8. Then it will recruit Smad4 and translocate to the nucleus to activate osteogenic transcription factors including Runx2 and Osterix [14,15]. BMP-2 may also activate Smad2/3 through BMPRIa, which is more commonly observed in embryonic and transformed cells, which suggests that BMP-2 signaling is associated with cell division and developmental processes [8]. The non-canonical MAPK branches, including ERK1/2, p38, and JNK, are also simultaneously activated by BMP-2. They will then converge on Runx2 to regulate osteoblastic differentiation. Inflammatory cytokines TNF-α and IL-1β, which are elevated in patients receiving exogenous BMP-2, also demonstrate the activation of the same p38 and ERK1/2 cascades but in an opposing direction, causing inhibition of BMP-2-induced Runx2 expression and osteoblastic differentiation [16,17]. 

The extracellular matrix (ECM) also influences cell behavior by controlling the amount, location, and timing of growth factor delivery to cell receptors. Positively charged BMP-2 will stick to the negatively charged chains of ECM proteoglycans, such as heparin, heparan sulfate, and chondroitin sulfate. Signaling is also limited by extracellular antagonists such as Noggin and intracellular inhibitory Smads, while membrane-associated proteins including CD44 and integrins may modulate receptor activation. BMP-2 may also be retained in extracellular complexes before release by matrix-processing enzymes, and receptor complexes are subsequently internalized and recycled [18].

### 3.3. Biological Rationale of BMP-2 in Bone Repair

BMP-2 currently serves as one of the most potent osteoinductive molecules involved in orthopedics. Recombinant human BMP-2 (rhBMP-2) has been widely investigated as a biological substitute for autologous bone grafts because it is capable of stimulating osteogenesis while avoiding donor-site morbidity [19,20]. In the United States, rhBMP-2/ACS (INFUSE^®^ Bone Graft, Medtronic, Minneapolis, MN, USA) is FDA-approved for single-level anterior lumbar interbody fusion with specified devices, acute open tibial shaft fractures treated with an intramedullary nail and within 14 days of injury, and certain oral–maxillofacial indications (sinus and alveolar ridge augmentation) [21]. 

The rationale for BMP-2 in fracture repair lies in its role in initiating fracture healing, driving osteogenic and chondrogenic differentiation, and inducing vascular responses. A previous study demonstrated that mice lacking BMP-2 in limb bones developed spontaneous fractures that never healed, and the earliest mechanism of fracture healing appeared to be blocked in these circumstances [22]. Furthermore, other osteogenic stimuli within the limb bones cannot compensate for the absence of BMP-2. This demonstrated that BMP-2 can be identified as an independent and non-redundant endogenous mediator of fracture repair [23].

During osteoclast-mediated degradation in fracture healing, BMP-2 is released from bone matrix and acts as a chemotactic signal to recruit mesenchymal stem cells (MSCs) to the fracture site [24]. This molecule plays a central part in the regulatory mechanism of all three major stages of fracture healing, the inflammatory response, chondrogenic phase, and osteogenic phase [21]. Exogenous BMP-2 has also been shown to increase deposition and resorption of both cartilage and bone during endochondral ossification. It was found to be associated with the stimulation of osteoclastogenesis but not angiogenesis in the early repair phase [23].

BMP-2 delivery also enhances neovascularization in critical-sized bone defects. However, this effect appears to happen via indirect paracrine signaling rather than direct endothelial stimulation. A study with conditioned medium from BMP-2-treated mesenchymal progenitors and osteoblasts showed the induction of endothelial cell migration, confirming that osteoprogenitor cells mediate the angiogenic response [25].

## 4. Current Clinical Applications of BMP-2

### 4.1. Application in Acute Fractures

The randomized controlled BESTT trial demonstrated that treatment with rhBMP-2 was associated with a 44% reduction in the need for secondary interventions, accelerated fracture healing, and lower infection rates compared with standard intramedullary nail fixation in open tibial fractures [4]. These findings were further supported by the meta-analysis conducted by Wei et al., which showed that the adjunctive use of rhBMP-2 with intramedullary nail fixation significantly reduced both secondary intervention rates and treatment failure in patients with open tibial shaft fractures. However, the effect of rhBMP-2 on infection risk remains controversial. The BESTT trial reported lower infection rates in the rhBMP-2/absorbable collagen sponge (ACS) group, potentially attributable to rhBMP-2–induced osteogenesis and enhanced local vascularization. In contrast, the RCT by Aro et al. reported a higher infection rate in the rhBMP-2/ACS group compared to standard of care [26]. Nevertheless, evidence from meta-analyses suggests that timely and adequate soft tissue management remains the most critical determinant in preventing infection, as infection rates were equally high in both treatment groups (21% in the rhBMP-2/ACS group versus 19% in the standard-of-care group) [27]. 

A post hoc subgroup analysis pooling data from two RCTs (BESTT and Aro et al.), which included patients with the most severe open tibial fractures (Gustilo–Anderson type IIIA and IIIB), identified this population as the most cost-effective target for rhBMP-2 therapy. The addition of rhBMP-2 to standard treatment significantly reduced the need for bone grafting procedures and other secondary interventions, resulting in improved clinical outcomes. Furthermore, a subsequent economic analysis based on the BESTT trial demonstrated that restricting rhBMP-2 use to patients with type IIIA and IIIB open tibial fractures was associated with overall cost savings [28]. A multicenter prospective single-arm study by Sakong et al. evaluating 90 patients with acute traumatic upper and lower extremity fractures demonstrated that 96.2% of patients achieved radiographic union at 12 months using HA-based rhBMP-2 delivery, with only a 1.2% adverse event rate and no ectopic ossification. However, patients with open fractures still had significantly higher treatment failure compared to closed fractures in this study, with 20% and 3%, respectively, suggesting that HA-based rhBMP-2 alone may not be sufficient for all open fracture cases, especially in those with complex and systemic conditions that may inhibit osteogenesis process [29].

Table 1 summarizes the key clinical studies of rhBMP-2 use in orthopedic trauma and limb reconstruction.

### 4.2. Critical-Sized Bone Defects and Limb Reconstruction

Critical-sized bone defects, defined as bone defects that will not heal spontaneously without intervention, are still a significant challenge in orthopedic patients. There is no universally accepted size threshold for a critical-sized defect. In the tibia, defects longer than approximately 2 cm with more than 50% circumferential cortical loss are generally unlikely to heal without additional intervention [35]. Within this setting, rhBMP-2 is attractive as an adjunct because it may provide osteoinductive activity while potentially reducing dependence on large volumes of autologous bone. rhBMP-2 is considered a biological component of reconstruction, along with restoration of mechanical stability, vascularity, viable soft-tissue coverage, and appropriate defect architecture [36]. Several observational studies have reported favorable union with rhBMP-2 augmentation in bone defects, potentially reducing the volume of autograft required, but comparative evidence is limited and still inconsistent. Son et al. reported that compared to induced membrane reconstruction without BMP augmentation, the rhBMP-2/HA-augmented group was associated with greater regenerated bone density, improved corticalization, and shorter time to union, supporting a potential synergistic effect between the osteoinductive stimulus of rhBMP-2 and the biologically active induced membrane [33]. Another study by Choi et al. prospectively evaluating rhBMP-2 with hydroxyapatite and autologous cancellous bone in patients with long-bone found that union was achieved in 95.8% of patients at 6 months and 100% at 12 months. However, this was a single-arm series of 24 patients, and all patients also received autologous cancellous bone, causing the isolated efficacy of rhBMP-2 cannot be evaluated [34]. However, the multicenter randomized trial by Cannada et al. in open tibial fractures with critical-sized defects of 1–5 cm, comparing rhBMP-2 delivered on an absorbable collagen sponge with iliac crest autograft, reported that union was achieved in 58.3% of the rhBMP-2 group and 81.8% of the autograft group [30]. These findings suggest that rhBMP-2 should not be considered a substitute for autograft in all cases, but rather as an osteoinductive adjunct used in combination with an appropriate osteoconductive scaffold or graft material. It should be considered more as an autograft extender or biological enhancer, particularly when graft volume is limited or the local environment is biologically compromised [34]. A recent prospective study comes from Sakong et al., who evaluated an HA-based rhBMP-2 delivery system in a multicenter study of acute upper- and lower-extremity fractures with bone defects. Of 81 patients included in the full analysis, radiographic union was achieved in 81.5% of cases at 6 months and 96.2% at 12 months, with a 95.0% treatment success rate and only one reported device-related adverse event. However, this study had no control group, included heterogeneous fracture sites and severities, and only 18.6% of the fractures were open [29].

### 4.3. Delayed Union and Nonunion 

Successful treatment of delayed union and nonunion requires correction of both mechanical and biological problems, including stable fixation, nonviable tissue debridement, infection management, and osteogenic environment restoration. A pooled different BMPs and orthobiologics systematic review by Impieri et al. suggested that BMPs, mesenchymal stromal cells, and platelet-rich plasma may improve fracture healing. However, substantial heterogeneity in fracture characteristics, treatment protocols, and orthobiologic formulations limits definitive conclusions regarding the superiority of any specific agent [37]. Some available clinical studies specifically evaluating rhBMP-2 have reported promising results. Fuchs et al. evaluated 91 patients with long-bone nonunion, including 72 treated with rhBMP-2 and 19 receiving standard treatment without BMP. The rhBMP-2 group demonstrated a significantly higher overall union rate (89% vs. 47%) and a shorter time to union [HR 2.78 (95% CI: 1.4–5.6)], with a particularly significant difference observed in femoral and tibial nonunions. However, no significant difference was demonstrated for humeral nonunion [32]. 

A meta-analysis of five comparative cohort studies by Xie et al. involving 394 long-bone nonunions found no significant improvement in union rate or healing time when rhBMP was added to autologous bone graft compared with autologous graft alone [38]. Subsequently, another meta-analysis similarly found no significant overall difference between rhBMP-based treatment and autologous bone graft in healing rate [RR 1.04 (95% CI: 0.96–1.12)] or healing time [SMD −0.31 (95% CI: −0.76 to 0.14)]. Nevertheless, subgroup analysis of moderate-quality studies favored rhBMP for both union rate [RR 1.35 (95% CI: 1.17–1.56)] and healing time [SMD −0.65 (95% CI: −1.08 to −0.22)] [39]. Recently, a 2025 network meta-analysis of 15 RCTs involving 1286 patients with delayed union or nonunion reported that BMP treatment was associated with higher healing rates and shorter healing times compared to standard treatment, with similar adverse events. However, this analysis pooled different BMP formulations, including rhBMP-2 and rhBMP-7, as well as heterogeneous anatomical sites and treatment strategies [40]. Overall, current evidence suggests that rhBMP-2 may be a useful biological adjunct in selected long-bone delayed unions and nonunions, particularly when local biological capacity is compromised, but it should complement rather than replace adequate mechanical stabilization and appropriate grafting or reconstructive strategies. 

### 4.4. Osteomyelitis and Fracture-Related Infection

Osteomyelitis and fracture-related infection (FRI) present a particularly challenging environment for bone regeneration. Persistent infection promotes inflammation, tissue necrosis, impaired vascularity, and loss of viable bone, while radical debridement required for infection eradication may further enlarge the bone defect. Accordingly, rhBMP-2 does not eradicate infection, but it provides osteoinductive support during reconstruction. Clinical evidence supporting BMP augmentation in osteomyelitis and FRI remains limited but suggests that osteoinduction may remain effective after appropriate surgical management of infection. Haubruck et al. retrospectively evaluated 156 femoral and tibial nonunions treated with either rhBMP-2 or rhBMP-7, including both septic and aseptic cases. rhBMP-2 was associated with consolidation in infected nonunions [OR 61.06 (95% CI: 2.21–1688.48)] especially when incorporated into a two-stage treatment strategy [OR 12.10 (95% CI: 2.74–53.31)]; however, these estimates come from a retrospective, nonrandomized study, with a very wide confidence intervals [31]. In a prospective cohort of 24 patients with complex long-bone nonunion, seven patients had segmental bone defects resulting from FRI. Reconstruction used macro-/microporous hydroxyapatite-loaded rhBMP-2 combined with autologous cancellous bone, with a maximum rhBMP-2 dose of 6 mg. The overall cohort achieved union rates of 95.8% at 6 months and 100% at 12 months, with improvements in functional outcomes and no detected rhBMP-2 antibodies or treatment-related adverse effects [34]. Three clinical situations should be distinguished. In active infection, rhBMP-2 has no established role, and active infection at the operative site is a contraindication in the product labeling [41]. Most of the available evidence concerns infection that has been controlled during staged reconstruction, for example after radical debridement and a period of local and systemic antibiotic therapy, as in the two-stage subgroup reported by Haubruck et al. [31]. Patients with a history of previous FRI but no current signs of infection form a third group, in whom the residual risk of recurrence should be considered. None of these situations has been studied in adequately powered comparative trials.

### 4.5. Dose–Response and Delivery-Dependent Optimization of rhBMP-2 

The clinical efficacy of rhBMP-2 is influenced by both dose and delivery system. In the BESTT trial involving 450 patients with open tibial fractures, Govender et al. compared standard treatment with rhBMP-2/ACS at 0.75 mg/mL (6 mg total) or 1.50 mg/mL (12 mg total). The 1.50 mg/mL group demonstrated a 44% reduction in the risk of secondary intervention for delayed union, along with faster fracture healing. Thus, among the concentrations evaluated with ACS in this particular setting, 1.5 mg/mL represented the most effective regimen for this delivery system [4]. However, efficacy does not appear to increase proportionally with increasing dose. In a subsequent randomized phase II/III trial of 369 patients with closed tibial fractures, rhBMP-2 delivered using an injectable calcium phosphate matrix at either 1.0 or 2.0 mg/mL failed to shorten fracture healing time compared with standard treatment; median radiographic union occurred at 13.0 and 15.9 weeks, respectively, compared with 13.1 weeks with standard care [6].

Recent clinical studies suggest that effective osteoinduction may be achievable with considerably lower total doses when rhBMP-2 is delivered through more retentive ceramic carriers. In a multicenter prospective study of acute traumatic extremity fractures using an HA-based delivery system, the mean rhBMP-2 dose was only 1.10 ± 1.26 mg; 48.1% of patients received 0.5 mg and 42.0% received 1.0 mg, while only a small minority received 3 or 6 mg. Radiographic union reached 81.5% at 6 months and 96.2% at 12 months, with few device-related adverse events [29]. Similarly, Choi et al. used HA-carried rhBMP-2 combined with autologous cancellous bone for long-bone nonunion, with an average dose of 4.96 mg and a maximum permitted dose of 6 mg, achieving 95.8% union at 6 months and 100% at 12 months without observed rhBMP-2-related adverse effects. Importantly, neither study directly randomized patients according to rhBMP-2 dose; therefore, these findings cannot establish 0.5–1.0 mg or approximately 5 mg as an optimal dose, but they suggest that contemporary delivery systems can achieve favorable outcomes using substantially less protein than the historical 12 mg ACS regimen [34].

Recent preclinical studies further demonstrate that the relationship between rhBMP-2 dose and bone regeneration is non-linear and carrier-dependent. In a rabbit metaphyseal defect study, rhBMP-2 delivered through a moldable calcium phosphate carrier was compared at 0.04, 0.16, and 0.6 mg/cm^3^. Higher doses accelerated early bone formation, with the 0.6 mg/cm^3^ group demonstrating significantly greater early bone formation than autograft at 3 weeks. However, by later time points there were no significant quantitative differences between the rhBMP-2 groups and the carrier alone. Moreover, ectopic bone formation was observed particularly at higher doses [42]. Another recent animal study using β-TCP (beta-tricalcium phosphate) similarly demonstrated a dose-dependent response across 5, 10, and 20 μg rhBMP-2, with 20 μg producing the greatest bone formation within that specific model. These findings indicate that increasing the dose may improve osteogenesis up to an effective threshold, but further escalation may provide diminishing benefit while increasing unintended bone formation [43].

### 4.6. Our Clinical Experience

The following two cases are presented as illustrative vignettes only. They were treated as part of routine clinical care, were not part of a research protocol, and are not intended as evidence of efficacy. In our center, we treated two young adults with complex femoral reconstruction using rhBMP-2 as an adjunct therapy. Both patients had multiple factors associated with impaired healing and were treated within the constraints of Indonesia’s universal health coverage system, where routine access to rhBMP-2 is limited by cost. In both cases, rhBMP-2 (Novosis™) was reconstituted according to the manufacturer’s instructions, and placed directly at the osteotomy site, avoiding contact with neurovascular structures/soft tissues. Patients were followed up clinically and radiographically.

The first patient was a 25-year-old man with a three-year femoral shaft malunion, 4 cm leg-length discrepancy, chronic transfusion-dependent alpha-intermediate thalassemia, anemia, malnutrition, cortical thinning, and disuse osteoporosis. He underwent corrective oblique osteotomy at the center of rotation of angulation followed by plate fixation, with 1 mg rhBMP-2 applied at the osteotomy site. Leg-length discrepancy improved to 1 cm after correction. Early callus formation was observed at three months, followed by progressive bridging and consolidation at seven months, accompanied by improvement in alignment, gait, and pain (Figure 3).

The second patient was a 23-year-old man with oligotrophic femoral shaft nonunion after multiple previous operations, associated with residual deformity and a 5 cm leg-length discrepancy. This patient also had multiple comorbidities, such as active smoking, obesity, and coagulopathy. We performed staged reconstruction, initially using autologous cancellous grafting, corticotomy, and gradual lengthening with a limb reconstruction system. After union at the lengthening site, definitive fixation was performed using a distal femoral locking plate, and 0.5 mg rhBMP-2 was applied to a residual 1.5 cm defect. At six months, progressive callus bridging was present, pain and gait had shown much improvement, and the residual leg-length discrepancy was only 0.5 cm. Radiographic evaluation is presented in Figure 4.

Both cases illustrate a potential role for rhBMP-2 as a biological adjunct in selected patients with compromised healing biology when conventional reconstructive principles have already been addressed. They also highlight a practical issue relevant to resource-limited health systems, in which the potential clinical value of lower-dose strategies may be considered and warrants further evaluation while balancing cost considerations, uncertain dose–response relationships, and the absence of high-quality studies supporting dose reduction.

### 4.7. Clinical Decision-Making

Current evidence does not explicitly support routine rhBMP-2 use in all traumatic bone defects. The role is better considered when the expected biological benefit is sufficient to justify the additional cost and potential side effects. In acute fractures, the clearest benefit was reported in severe open tibial fractures, particularly Gustilo–Anderson type IIIA and IIIB injuries, where rhBMP-2 reduced secondary interventions in the BESTT program [4,28]. In patients with delayed union and nonunion, rhBMP-2 may be considered when mechanical stability has been restored but biological healing remains compromised. The available comparative evidence remains inconsistent, but autologous bone graft continues to be an important reference treatment [32,38,39,40]. In infected nonunion or fracture-related infection cases, rhBMP-2 should only be viewed as a reconstructive adjunct after managing the infection thoroughly rather than as the main treatment for infection itself [31,34].

Defect size, host biology, graft availability, soft-tissue condition, and the planned reconstruction should therefore guide its use. Carrier and dose are also clinically relevant factors, with newer hydroxyapatite-based systems having reported favorable union with lower total rhBMP-2 doses, but these studies have not established a universal optimal dose [29,34]. Therefore, treatment choice should apply the lowest effective dose supported by the selected delivery system, while avoiding uncontrolled local diffusion and ectopic bone formation.

## 5. BMP-2 Limitations, Safety, and Implementation Considerations

### 5.1. Evidence Limitations

The clinical evidence of rhBMP-2 application in orthopedic trauma remains heterogeneous. Available studies differ substantially in fracture type, defect size, host biology, fixation strategy, carrier, rhBMP-2 dose, and concomitant use of autologous graft. Most studies are small prospective or retrospective comparative series, while adequately powered randomized trials remain limited. Additionally, the evidence is also indication-dependent. Benefit was demonstrated in the BESTT trial for selected open tibial fractures, whereas subsequent trials using different fixation or delivery strategies did not consistently reproduce this effect [4,6,26]. Similarly, evidence in nonunion and critical-sized defects remains mixed, with favorable observational results but less consistent comparative evidence [32,34].

### 5.2. Safety and Adverse Events

Safety concerns remain an important limitation to widespread rhBMP-2 use. Some reported complications include local inflammation, wound complications, surgical-site infection, heterotopic or ectopic bone formation, and pseudarthrosis. A postmarketing surveillance study by Woo et al. identified 62 adverse event reports with rhBMP-2 use, with almost half of them requiring an additional intervention. However, no established direct causal relationship was found [5]. Additionally, clinical trials have also produced inconsistent findings regarding safety signals. The BESTT trial reported fewer infections with rhBMP-2, whereas Aro et al. reported a higher infection rate in the rhBMP-2 group [4,26]. Meta-analytic evidence has not demonstrated a consistent increase in adverse events, but interpretation is limited by differences in BMP formulation, indication, and reporting [27,38].

### 5.3. Dose-Dependent Complications

The relationship between rhBMP-2 dose and clinical response is not linear. Excess local exposure may also increase unintended osteogenic activity. Preclinical studies demonstrate greater early bone formation with increasing rhBMP-2 dose, but this benefit plateaus while ectopic bone formation becomes more prominent at higher concentrations [42,43]. Consequently, increasing dose should not be regarded as a simple strategy to overcome poor biological conditions. The main treatment objective is likely the lowest dose that maintains sufficient local osteoinductive activity within a given delivery system.

### 5.4. Carrier-Related Limitations

Carrier characteristics are inseparable from rhBMP-2 dose and biological effect. The absorbable collagen sponge (ACS) was the most commonly used carrier in early clinical trials; it delivers rhBMP-2 at relatively high concentrations, but provides limited control over protein retention and release [4]. By comparison, HA-based systems have recently been developed and have achieved favorable outcomes using substantially lower doses [29,34]. However, no currently available studies were designed to directly compare carriers or determine dose equivalence. 

The failure of rhBMP-2 delivered through an injectable calcium phosphate matrix to improve healing in closed tibial fractures further proved that the efficacy cannot be attributed to protein dose alone [6]. The efficacy and adverse effects may be altered by some factors, including differences in protein binding, release kinetics, scaffold architecture, local retention, and osteoconductive properties. Accordingly, evidence generated with one carrier–dose combination should not be directly generalized to another.

### 5.5. Cost and Implementation

The application of rhBMP-2 was correlated with high cost, which remains an important barrier to routine use in clinical practice. In non-complicated fractures or settings in which rhBMP-2 provides little additional clinical benefit, its additional cost is difficult to justify. However, the cost may be justified in selected high-risk cases, which often necessitate repeated interventions to achieve bone union and reduce complications. The initial financial burden of rhBMP-2 may be deemed cost-ineffective at first, but the prevention of further multiple reoperations, shorter hospital stay, and minimized cost associated with prolonged disability may justify the use of rhBMP-2 in selected high-risk cases. Further studies should evaluate which populations and cases are most likely to benefit from rhBMP-2, as well as their cost-effectiveness and determine whether its clinical benefits justify the associated costs. The cost-effectiveness is more likely to depend on selective use in patients with substantial biological compromise such as in complex fractures, nonunion, and critical-sized bone defects with a high expected risk of repeated reconstruction. 

### 5.6. Regulatory and Off-Label Considerations

In the United States, the approved trauma indication for INFUSE Bone Graft is acute open tibial shaft fracture in skeletally mature patients treated with intramedullary nail fixation after appropriate wound management, with application within 14 days of the initial fracture. However, many applications discussed in this review, including established nonunion, critical-sized defects, infected nonunion, and other forms of complex limb reconstruction, extend beyond this specific trauma indication [41]. Currently, rhBMP-2 use is not covered by Indonesia’s Universal Health Coverage (BPJS) and its use in the indications discussed here is off-label. Further studies are warranted to identify the patient populations most likely to benefit.

## 6. Emerging Directions and Future Opportunities

### 6.1. Carrier Optimization and Dose Reduction via Controlled and Sustained Local Delivery

Current development of rhBMP-2 is increasingly focused on improving local retention and release kinetics. Conventional absorbable collagen sponge carriers cause rapid burst release and uncontrolled diffusion of rhBMP-2, causing the need for higher doses and driving adverse outcomes. Hydrogels, microparticles, mineral-based scaffolds, and affinity-based systems such as heparin-containing carriers may prolong local BMP-2 activity, while reducing the burst release and diffusion into surrounding tissues [24]. A hybrid nanofiber mesh/alginate delivery system achieved 2.5-fold greater bone volume than collagen sponge at the same 1.0 μg dose via better protein retention [44]. A preclinical study by Kim et al. using calcium-binding PLGA microparticles releasing rhBMP-2 scaffolds reported a sustained release of rhBMP-2 showing gradual stimulation of bone growth while avoiding excessive bone formation at non-targeted sites, using the particles’ higher surface area-to-volume ratios and bone-targeting coatings that promote targeted adhesion at bone defect sites [45]. Other innovative delivery system alternatives for a sustained release of rhBMP-2 are injectable hydrogels based on temperature-responsive polymers and 3D-printed scaffolds composed of polycaprolactone (PCL) and β-tricalcium phosphate (β-TCP) which have shown supportive properties in bone tissue growth [46,47].

Preclinical carrier comparisons suggest that material properties influence the effective dose. Fibrin matrix achieved equivalent bone union to collagen sponge at a sevenfold lower BMP-2 dose, with fivefold higher cumulative release during the observation period. Biphasic calcium phosphate with a layer-by-layer collagen/heparin coating reduced initial burst release by over 50% and induced significantly more new bone at an extremely low 0.01 mg/mL BMP-2 concentration versus conventional soaking. These observations show the importance of the BMP-2 delivery system, more specifically how it is released and avoids local burst diffusion.

### 6.2. Composite Osteogenic-Angiogenic Strategies

Large bone defects require both osteogenesis and adequate vascularization. BMP-2 may promote neovascularization indirectly through osteoprogenitor-mediated signaling, but this effect may be insufficient in severely compromised defects [25]. The combination delivery of BMP-2 with angiogenic factors such as VEGF is being explored to provide coordinated vascular and osteogenic stimulation. Future strategies should focus not only on factor combination, but also on appropriate timing and sequence of release to better reproduce physiological fracture healing. Alarcin et al. reported that BMP-2, alginate, endothelial cells, and vascular endothelial growth factor (VEGF)-loaded PLGA nanoparticle 3D bioprinting enabled sustained release of growth factor (GF), thus stimulating osteogenic and angiogenic processes in an in vivo model [48]. In a Sprague-Dawley rat model, VEGF with BMP-2 on a PLGA composite also demonstrated an increase in supportive vascular network formation [49].

### 6.3. BMP-2 in Infected Bone Defects

In fracture-related infection, rhBMP-2 may support bone regeneration after adequate debridement and infection control, but it does not primarily have an antimicrobial role. The emerging multifunctional scaffolds aim to combine antibacterial activity with subsequent osteogenic stimulation, potentially addressing infection control and bone regeneration within the same reconstructive strategy. However, future research is still warranted because whether these approaches improve both eradication of infection and union remains unknown. Recent promising strategies include the experimental antibacterial enzymes or antibiotics incorporated within BMP-2 hydrogel systems, such as gelatin methacryloyl (GelMA) and carboxymethyl chitosan (CMCS) composite, recombinant lysostaphin, or sulfated chitosan. In preclinical animal studies, these hydrogels have shown promising results in eradicating infection in infected bone defects while still promoting bone formation [50,51,52].

### 6.4. Gene- and mRNA-Based BMP-2 Delivery

Gene- and mRNA-based approaches may allow for local production of BMP-2 without the high protein concentrations required with conventional recombinant delivery. Chemically modified mRNA encoding BMP-2 has shown promising preclinical results, including bridging of critical-sized defects and restoration of mechanical strength with less excessive callus formation than recombinant protein. In a rat femoral segmental defect model, 50 μg of BMP-2 cmRNA delivered in a fibrin gel achieved complete defect bridging by four weeks, with faster mechanical strength recovery and earlier remodeling than rhBMP-2 [53]. BMP-2 mRNA delivered via lipid nanoparticles in collagen sponge also demonstrated superior bone regeneration with reduced callus formation compared to rhBMP-2 in a rat femur model [54]. However, some concerns, including delivery efficiency, duration of expression, immunogenicity, manufacturing complexity, and regulatory requirements, remain limitations to clinical translation.

### 6.5. Patient-Specific and Precision Orthobiologic Strategies

The variable response to rhBMP-2 across clinical settings suggests that treatment should be tailored to both the defect and the host. Diabetes and osteoporosis impair bone regeneration and carrier modifications may be needed to maintain local rhBMP-2 bioactivity in these conditions. Experimental data indicate that higher rhBMP-2 doses may partially compensate for impaired diabetic healing, whereas slow-release ceramic or hydrogel composites improve graft stability in osteoporotic bone [55]. In aged bone, rhBMP-2 exacerbates the osteogenesis–adipogenesis imbalance, driving excess adipocyte accumulation that suppresses osteogenic differentiation [7]. Other factors also need to be considered, including defect size and geometry, vascularity, infection status, and fixation stability. Future strategies may therefore combine patient selection, carrier choice, dose, and scaffold design rather than applying a uniform protocol across different reconstructive problems.

### 6.6. Priorities for Future Clinical Trials

Future multicenter randomized controlled trials stratifying patients by defect size, anatomical site, host status, infection status, and fixation stability, while clearly reporting carrier type, absolute dose, concentration, delivery method, and concurrent graft use are warranted. Outcomes should be standardized to include union rate, time to union, reoperation, function, adverse events, infection or reinfection, and cost. Adequately powered multicenter trials comparing clinically relevant carrier–dose and graft combinations are needed to determine when rhBMP-2 provides meaningful additional clinical and economic benefit.

## 7. Conclusions

rhBMP-2 has demonstrated promising osteoinductive properties as an adjunct in orthopedic trauma and limb reconstruction strategies, particularly in the management of complex fractures, nonunion, and critical-sized bone defects. However, its clinical efficacy remains inconsistent across anatomical sites, indications, and patient conditions, as its efficacy depends on the interaction between rhBMP-2 and patient-related factors, local mechanical and biological environment, delivery systems, and the dosage regimen. Current evidence does not support the routine use of rhBMP-2 across all indications in orthopedic trauma and limb reconstruction. Further randomized controlled trials are needed and should prioritize stratified patient selection to identify the populations most likely to benefit from the osteoinductive properties of rhBMP-2. Comparative studies between carrier systems that provide controlled and sustained release, as well as studies establishing the minimum effective dose, are also required to improve osteoinductive properties while reducing treatment-related complications.

## Figures and Tables

**Figure 1:**
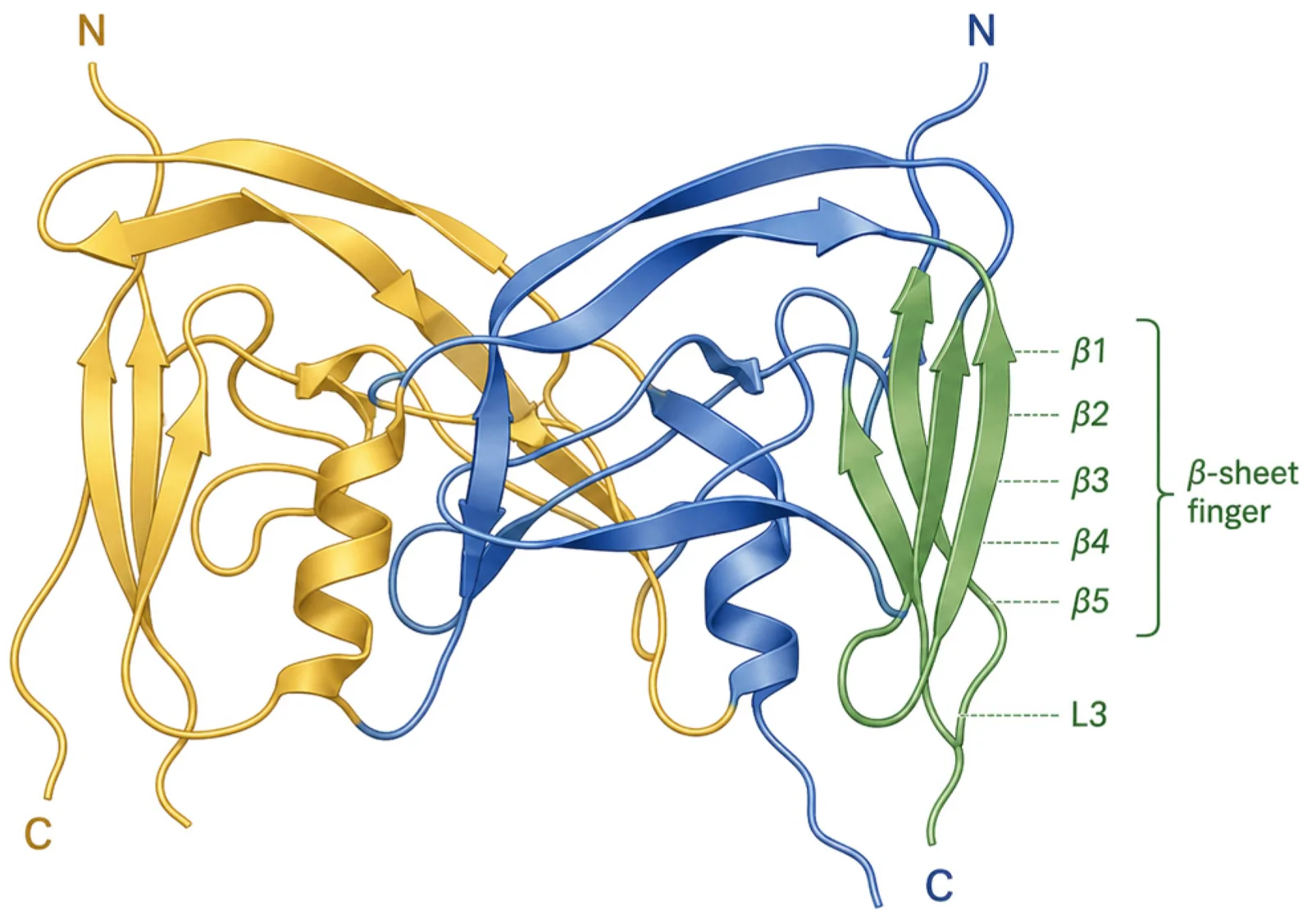
BMP Structure.

**Figure 2:**
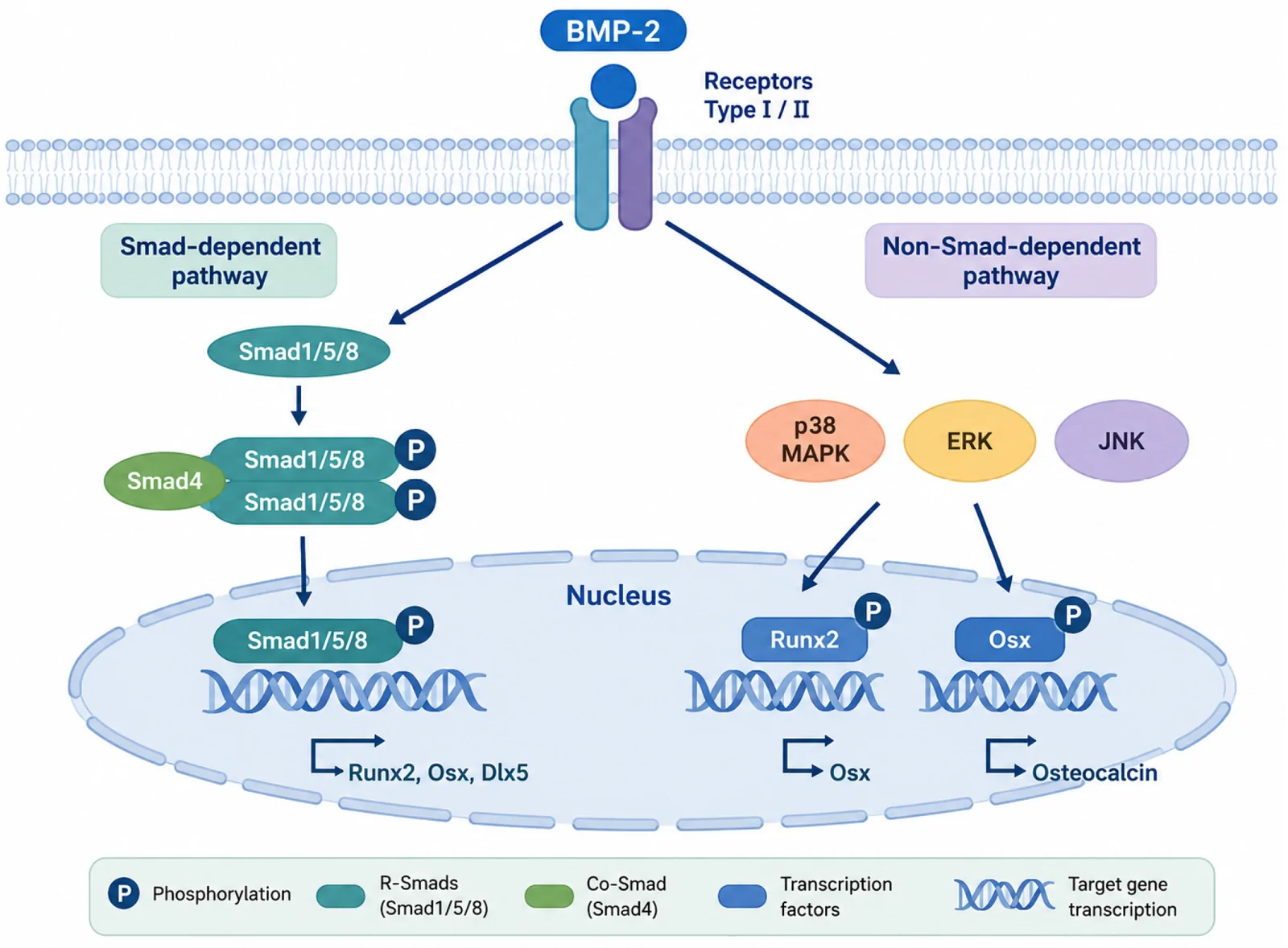
BMP signaling pathway.

**Figure 3:**
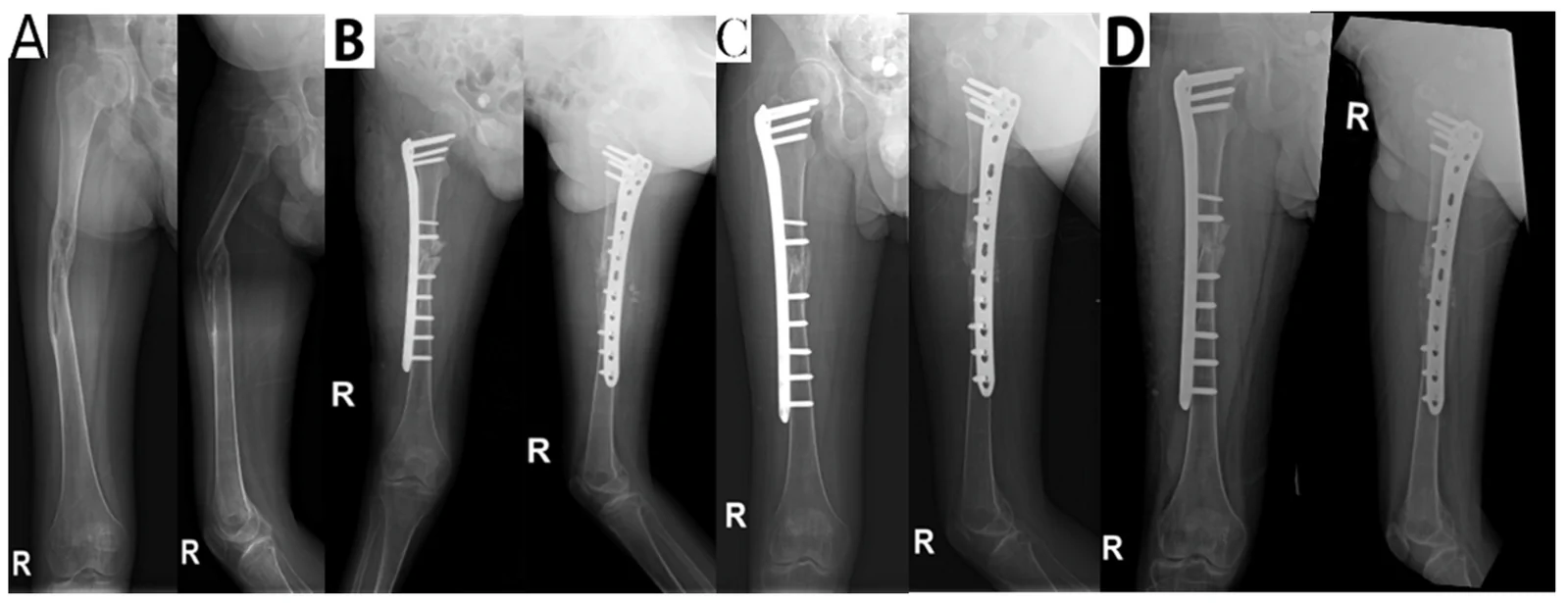
Case 1: 25-year-old man with a 3-year femoral shaft malunion, 4 cm leg-length discrepancy, and transfusion-dependent thalassemia. (**A**) Preoperative anteroposterior (AP) and lateral radiographs; (**B**) immediate postoperative radiographs after corrective oblique osteotomy, plate fixation, and application of 1 mg rhBMP-2 at the osteotomy site; (**C**) 3 months postoperatively, showing early callus formation; (**D**) 7 months postoperatively, showing progressive bridging callus and consolidation.

**Figure 4:**
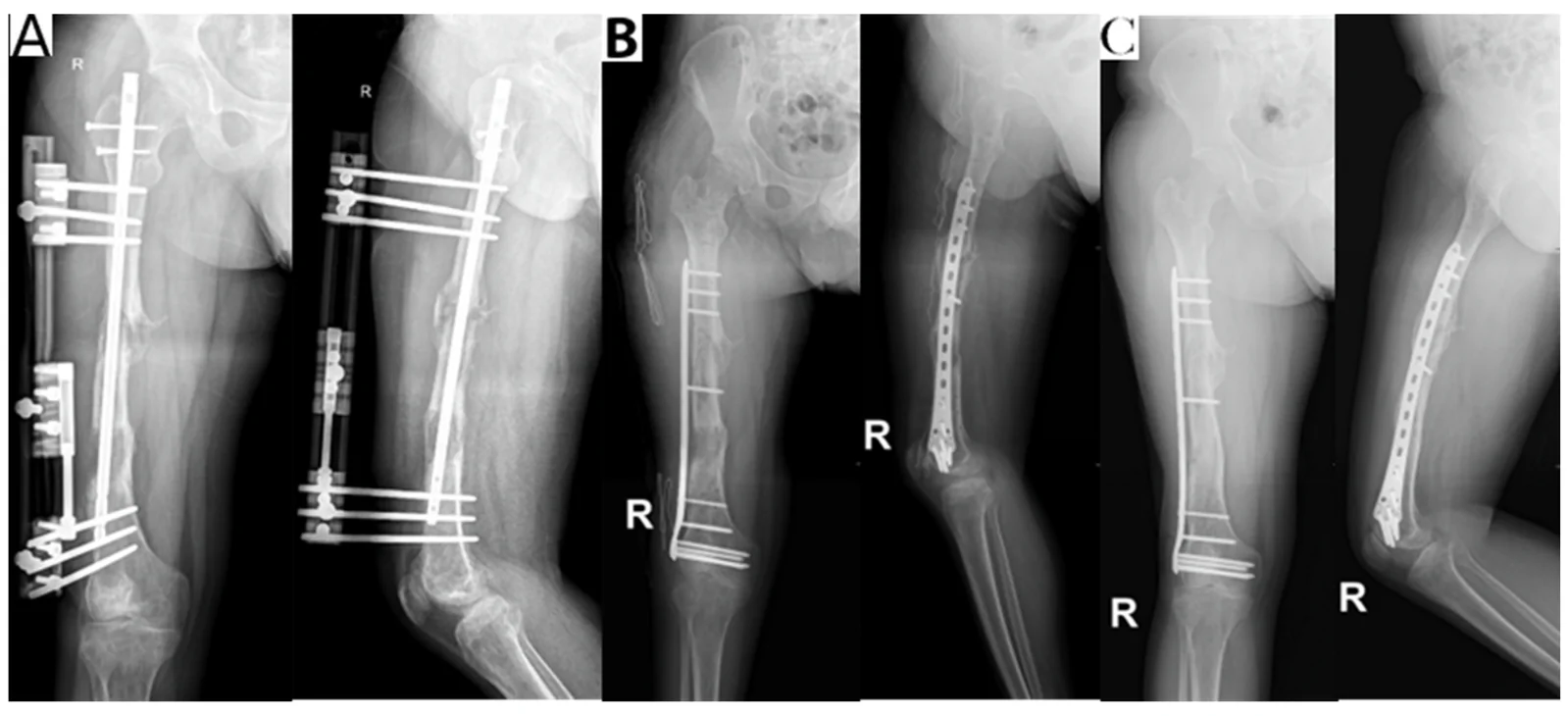
Case 2: 23-year-old man with oligotrophic femoral shaft nonunion after multiple previous operations. (**A**) Preoperative radiographs showing nonunion, residual deformity, and 5 cm leg-length discrepancy; (**B**) after definitive distal femoral locking plate fixation with 0.5 mg rhBMP-2 applied to a residual 1.5 cm defect; (**C**) 6 months after definitive fixation, showing progressive callus bridging.

**Table 1:** Key Clinical Evidence for rhBMP-2 in Orthopedic Trauma and Limb Reconstruction.

Study	Year	Design/*n*	Level of Evidence	Indication	Comparator	Carrier	rhBMP-2 Dose	Primary Outcome	Main Finding	Major Limitation
**Acute fracture**										
Govender et al. (BESTT) [4]	2002	Prospective randomized controlled trial (*n* = 450)	I	Acute open tibial shaft fractures	Standard care with IMN fixation	Absorbable collagen sponge (ACS)	0.75 mg/mL (6 mg) or 1.50 mg/mL (12 mg)	Secondary intervention for delayed union/nonunion within 12 months	The 1.50 mg/mL group reduced the risk of secondary intervention by 44% and showed faster fracture healing; the lower dose did not show the same benefit.	Historical trial; the applicability to contemporary reamed fixation and current soft-tissue protocols is uncertain.
Aro et al. [26]	2011	Multicenter randomized controlled trial (*n* = 277)	I	Acute open tibial fractures treated with reamed IM nailing	Standard care with IMN fixation	Absorbable collagen sponge (ACS)	1.50 mg/mL (12 mg)	Clinical and radiographic healing at 13 and 20 weeks	Addition of rhBMP-2 did not significantly accelerate healing; secondary procedures were similar, while infection was numerically higher with rhBMP-2 (19% vs. 11%).	Only 13% of fractures were Gustilo–Anderson IIIB; limited inference for the most severe open injuries.
Cannada et al. (METRC) [30]	2019	Multicenter randomized controlled trial (*n* = 30)	II	Open tibial fractures with critical-sized bone defects	Iliac crest autograft (ICBG)	ACS with allograft	Not reported	Radiographic union within 52 weeks	Union occurred in 58.3% of evaluable rhBMP-2 patients vs. 81.8% with ICBG; equivalence was not demonstrated.	Very small trial and only 23/30 patients had 52-week union data, limiting power for an equivalence conclusion.
Lyon et al. [6]	2013	Double-blind randomized phase II/III trial (*n* = 369)	I	Acute closed tibial diaphyseal fractures	Standard care ± carrier control	Injectable calcium phosphate matrix (CPM)	1.0 or 2.0 mg/mL	Time to fracture union and return to normal function	Neither dose shortened fracture healing compared with standard care; median radiographic union was 13.0 (1.0 mg/mL) and 15.9 (2.0 mg/mL) weeks vs. 13.1 weeks with standard care.	Closed fractures have relatively favorable baseline biology; findings may not extrapolate to high-risk open fractures or nonunion.
Sakong et al. [29]	2026	Prospective multicenter single-arm study (*n* = 90 enrolled, 81 analyzed)	IV	Acute upper- and lower-extremity fractures with bone defects	None	HA-based system (Novosis™)	Mean 1.10 ± 1.26 mg (mainly 0.5–1.0 mg)	Radiographic union at 6 and 12 months	Union was 81.5% at 6 months and 96.2% at 12 months; treatment success was 95.0%, with one device-related adverse event.	No control group, heterogeneous fracture sites and severities, and no dose randomization; only 18.6% were open fractures.
**Non-union and bone defects**										
Haubruck et al. [31]	2018	Retrospective comparative study (*n* = 156)	III	Femoral and tibial nonunion, including septic nonunion	rhBMP-7	Not clearly standardized/reported	Not reported	Radiographic consolidation	Consolidation was higher with rhBMP-2 than rhBMP-7 (91% vs. 58%); association was particularly strong in infected nonunion and two-stage reconstruction.	Retrospective, nonrandomized treatment allocation, heterogeneous one- and two-stage strategies, and very wide confidence intervals.
Fuchs et al. [32]	2021	Prospective single-center open-label comparative study (*n* = 91)	II	Long-bone nonunion	Standard nonunion surgery without BMP	ACS (InductOs) + iliac crest autograft	12 mg	Union rate and time to union	Higher union rate (89% vs. 47%) and faster healing with rhBMP-2; benefit was most apparent in femoral and tibial nonunions.	Nonrandomized study; allocation was partly influenced by insurance reimbursement, with markedly unequal group sizes.
Son et al. [33]	2022	Retrospective propensity score-matched study (*n* = 28)	III	Critical-sized bone defects treated with induced membrane technique (IMT)	IMT without rhBMP-2	Hydroxyapatite (HA)	Not reported	CT-based quality and quantity of regenerated bone	rhBMP-2/HA improved bone density and corticalization and shortened time to union (316 vs. 585 days); overall union rate was not significantly different.	Small retrospective single-center cohort. The union rate difference was not statistically significant despite favorable imaging endpoints.
Choi et al. [34]	2024	Prospective single-arm case series (*n* = 24)	IV	Long-bone nonunion with bone defects, including FRI-related defects	None	Macro-/microporous HA granules + autologous cancellous bone	Mean 4.96 mg	Radiographic union and safety	Union reached 95.8% at 6 months and 100% at 12 months, with no detected BMP-2 antibodies or treatment-related adverse effects.	Small uncontrolled cohort. The concomitant autologous bone prevents attribution of the outcome to rhBMP-2 alone.

Level of evidence was graded according to the *Journal of Bone and Joint Surgery* levels of evidence for therapeutic studies: I, high-quality randomized controlled trial; II, lesser-quality randomized controlled trial (e.g., <80% follow-up or early termination) or prospective comparative study; III, retrospective comparative study; IV, case series without a concurrent control group.

## Data Availability

The original contributions presented in this study are included in the article. Further inquiries can be directed to the corresponding author.

## Abbreviations

The following abbreviations are used in this manuscript:

BMP	Bone morphogenetic protein
BMPR	Bone morphogenetic protein receptor
β-TCP	β-tricalcium phosphate
CI	Confidence interval
ECM	Extracellular matrix
ERK	Extracellular signal-regulated kinase
FDA	U.S. Food and Drug Administration
FRI	Fracture-related infection
HA	Hydroxyapatite
IL	Interleukin
IMT	Induced membrane technique
IMN	Intramedullary nail
JNK	c-Jun N-terminal kinase
MAPK	Mitogen-activated protein kinase
mRNA	Messenger RNA
MSC	Mesenchymal stem cell
ODI	Oswestry Disability Index
OR	Odds ratio
RCT	Randomized controlled trial
rhBMP-2	Recombinant human bone morphogenetic protein-2
RR	Risk ratio
TGF-β	Transforming growth factor-β
